# Nomogram to predict prognosis of head and neck rhabdomyosarcoma patients in children and adolescents

**DOI:** 10.3389/fonc.2024.1378251

**Published:** 2024-03-25

**Authors:** Jinwen Wu, Qi Zeng

**Affiliations:** ^1^ Department of Ultrasound, The First Affiliated Hospital of Gannan Medical University, Ganzhou, Jiangxi, China; ^2^ Gannan Medical University, Ganzhou, Jiangxi, China

**Keywords:** rhabdomyosarcoma, head and neck, prognostic survival analysis, nomogram, SEER

## Abstract

**Purpose:**

This study aims to explore the prognostic factors of head and neck rhabdomyosarcoma (HNRMS) in children and adolescents and construct a simple but reliable nomogram model for estimating overall survival (OS) of patients.

**Methods:**

Data of all HNRMS patients during 2004–2018 were identified from the Surveillance, Epidemiology, and End Result database. Kaplan–Meier method was performed to calculate OS stratified by subgroups and comparison between subgroups was completed by log-rank test. Univariate and multivariate Cox regressions analysis were employed for identifying independent predictors, which subsequently were used for a predictive model by R software, and the efficacy of the model was evaluated by applying receiver operating curve (ROC), calibration and decision curve analysis (DCA).

**Results:**

A total of 446 patients were included in the study. The 1-, 3-, and 5-year OS rate of the whole cohort was 90.6%, 80.0%, and 75.5%, respectively. The results of univariate and multivariate Cox regression analysis indicated that the primary site in parameningeal region, alveolar RMS histology, M1 stage, IRS stage 4, surgery, and chemotherapy were significant prognostic factors (all *P*<0.05). The performance of nomogram model was validated by discrimination and calibration, with AUC values of 1, 3, and 5 years OS of 0.843, 0.851, and 0.890, respectively.

**Conclusion:**

We constructed a prognostic nomogram model for predicting the OS in HNRMS patients in children and adolescents and this model presented practical and applicable clinical value to predict survival when choosing treatment strategies.

## Graphical Abstract

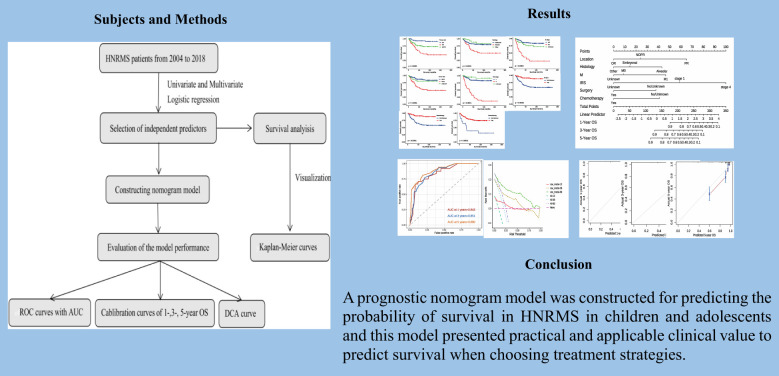

## Introduction

Rhabdomyosarcoma (RMS) is a rare but aggressive malignant tumor originating from the embryonic mesenchyme of the rhabdomyosarcoma tissue (1) and is the most common soft tissue sarcoma (STS) in children and adolescents (2), accounting for about 4%–5% of cancers in the pediatric population (3). Although RMS can occur anywhere in the body, 40% of primary lesions originate in the head and neck region that can be further classified as the orbital RMS (OR), parameningeal RMS (PR) and non-orbital-parameningeal RMS (NOPR) (4–6). RMS is characterized by unclear prognostic factors and poor survival, it is reported that high-risk RMS and recurrent disease have 5-year survival rates of lessen 30% and 17%, respectively (7). However, the overall survival (OS) rate of patients has been increased through better staging, risk grouping, improved localized treatments, and better supportive care after The Intergroup Rhabdomyosarcoma Study (IRS) was established in the United States in 1972 (8, 9). The treatment method of RMS is multimodal, involves multi-agent systemic chemotherapy, along with surgical resection of the tumor with or without addition of the radiation therapy for control of local lesion (10). The identification of prognostic factors for RMS may help with optimizing treatment protocols.

A nomogram is a graphical tool for displaying the predicted value of individual survival based on significant variables produced by multivariate regression analysis briefly and intuitively (11, 12); currently, it has been widely used in disease prediction of malignant tumors such as laryngeal carcinoma and lung cancer etc (13–15). There are also some nomograms that have been constructed and proven to be beneficial in the management of RMS (16–18), and they present obvious advantages such as improved predictive accuracy, robustness, and usability, all of which increase their potentials in clinical practices. As a result, nomograms have been proposed as alternative methods, or perhaps new standard, to guide the management of cancer patients (19, 20). However, there is still a lack of nomogram model to predict the survival probability of head and neck rhabdomyosarcoma (HNRMS) patients in children and adolescents, which may be attributed to limited cases reports and small case studies in each single institution.

In this study, we analyzed the demographic, clinical characteristics and treatment strategies for predicting OS of HNRMS patients in children and adolescence by using population-based data recorded in the Surveillance, Epidemiology, and End Results (SEER), with the purpose of providing clinicians and patients with a simple but effective clinical tool to predict survival.

## Patients and methods

### Data collection

SEER-State software (version 8.4.2; http://seer.cancer.gov) was used to download RMS data from SEER database. Inclusion criteria were as follows: (1) primary tumor location in head and neck (anatomical codes: C00.0-C14.0, C 44.1-C44.4, C49.0, C69.0-C72.0); (2) year of diagnosis from 2004 to 2018; (3) age at diagnosis under 18 years old (4) histologic types were RMS (ICD-O-3 codes: 8900/3, 8901/3, 8902/3, 8910/3, 8912/3, 8920/3, and 8921/3). Exclusion criteria were listed as follows: (1) diagnosis made by autopsy or death certificate rather than pathohistological biopsy; (2) incomplete or unknown survival information. The original data for this study were obtained from publicly available database, and the present study complies with the principles of the Declaration of Helsinki. As such, this study was exempt from ethics review by the ethics committee.

### Variable definitions and end point

Variable definitions and information extracted from SEER database included patient ID, gender, age at diagnosis, primary sites, histology, TNM stage, tumor size, surgery, radiotherapy, and chemotherapy. Tumor primary site was classified into OR, PR, and NOPR; PR includes nasopharynx, nasal cavity, parapharyngeal region, sinuses, pterygopalatine fossa, middle ear, and mastoid process, while NOPR mainly consists of tongue, palate, and all other head and neck regions except obtial region (21). Histological types were classified into embryonal, alveolar, and other types. Tumor size was classified as ≤5 cm and >5 cm. RMS staging was based on the pre-treatment IRS staging system, which was shown in **Table 1**. OS was defined as the number of survival months that HNRMS patients to death from any cause at the end of follow-up, and the end point event was standardized by death from any cause, if not, censored was recorded.

**Table 1:** IRS staging system of rhabdomyosarcoma patients before treatment.

IRS stage	Primary site	T stage	Size(cm)	N stage	M stage
1	A	T1 or T2	≤5 or >5	N0, N1	M0
2	B	T1 or T2	≤5	N0	M0
3	B	T1 or T2	≤5 or >5	N0, N1	M0
4	A or B	T1 or T2	≤5 or >5	N0	M1

A refers to primary site in head and neck with favorable prognosis (OR or NOPR) and B refers to unfavorable prognosis (PR). T stage includes T1 and T2, T1: tumor was confined to primary organs or tissues; T2: tumor involves contiguous organs or structures. N stage includes N0 and N1, N0: no clinical or radiographic evidence of involvement of regional lymph nodes; N1: clinical or radiographic evidence of regional lymph node involvement. M stage includes M0 and M1, M0: no distant metastases on clinical, radiographic, or bone marrow assessment, M1: evidence of distant metastasis.

### Statistical analysis

Statistical analysis and graphs were finished by the R software (version 4.1.3; http://www.rproject.org). Continuous variables were expressed as median with interquartile range, while categorical variables were expressed as number (percentage). Variables selected by SEER database were preliminarily analyzed by univariate Cox regression, and variables with *P* < 0.05 were incorporated into multivariate Cox regression for identifying independent prognostic factors. The Kaplan–Meier method was performed to compare the OS. A nomogram prediction model was constructed based on by the results of multivariate Cox regression analysis to predict 1-, 3-, and 5-year OS of HNRMS in children and adolescents. The Bootstrap 1000 resampling method was used for calibration analysis of the nomogram. The discrimination ability was evaluated by receiver operating characteristic (ROC) with area under the curve (AUC), and the consistency was evaluated by calibration curves. Decision curve analysis (DCA) was used for evaluating clinical feasibility. The *P* < 0.05 was considered as statistical significance.

## Results

### Demographic and clinical characteristics of patients

According to the inclusion and exclusion criteria, 446 patients with HNRMS in children and adolescents were identified between 2004 and 2018 from the SEER database. Tumor primary site were OR in 110 cases (24.6%), PR in 82 cases (18.4%), and NOPR in 254 cases (57.0%). The embryonal was the most common histological type accounting for 65.9%, followed by the alveolar (22.7%), and other types (11.4%). As for the RMS staging:T1 (23.8%) and T2 (38.3%); N0 (60.8%) and N1(10.1%); M0 (59.9%) and M1 (14.3%); 53.1% of tumor size was less than 5 cm and 29.4% of which was more than 5 cm; patients were 309 cases (69.3%) in IRS stage 1, 66 cases (14.8%) in stage 4. In terms of treatment, the majority of patients received surgical treatment (94.4%) and more than half of patients received chemotherapy (64.8%), while less than half of patients received radiotherapy (26.0%). The demographics and clinical characteristics were shown in **Table 2**.

**Table 2:** Patient demographics and clinical characteristics.

Characteristics	Category	Number of cases	F (%)/M (P25, P75)
Gender			
	male	238	53.4
	female	208	46.6
Age			6 (4, 10)
Primary site			
	OR	110	24.6
	PR	82	18.4
	NOPR	254	57.0
Histology			
	Embryonal	294	65.9
	Alveolar	101	22.7
	Other	51	11.4
T stage			
	T1	106	23.8
	T2	171	38.3
	Unknown	169	37.9
N stage			
	N0	271	60.8
	N1	45	10.1
	Unknown	130	29.1
M stage			
	M0	267	59.9
	M1	64	14.3
	Unknown	115	25.8
Tumor size (cm)			
	≤5cm	237	53.1
	>5cm	131	29.4
	Unknown	78	17.5
IRS stage	1	309	69.3
	4	66	14.8
	Unknown	71	15.9
Surgery			
	Yes	421	94.4
	No/Unknown	25	5.6
Radiotherapy			
	Yes	116	26.0
	No/Unknown	330	74.0
Chemotherapy			
	Yes	289	64.8
	No/Unknown	157	35.2

### Survival analysis of different prognosis

**Figure 1:**
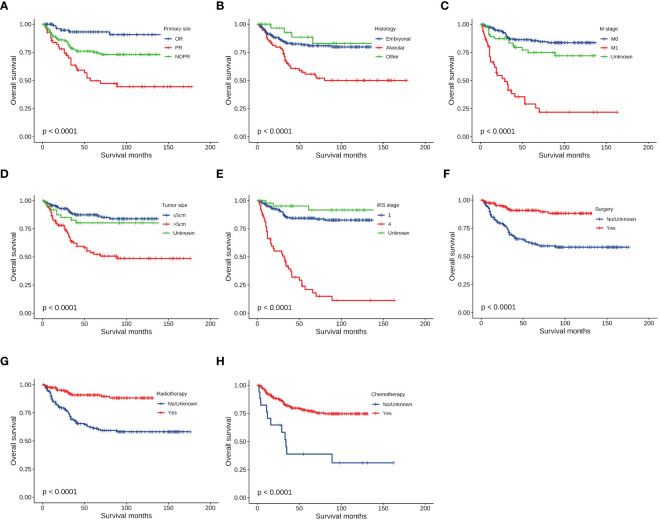
Kaplan-Meier curves of overall survival stratified by **(A)** Primary site, **(B)** Histology, **(C)** M stage, **(D)** Tumor size, **(E)** IRS stage, **(F)** Surgery, **(G)** Radiotherapy, **(H)** Chemotherapy.

The median follow-up time for the entire cohort was 54 months (interquartile range: 22–111 months). Less than half of patients (102, 22.8%) died during the follow-up time, and the 1-, 3-, and 5-year OS of patients were 90.6%, 80.0%, and 75.5%, respectively. To identify prognostic factors associated with OS in patients with HNRMS in children and adolescents, we conducted univariate and multivariate Cox regression analysis (**Table 3**). The results of univariate Cox regression analysis showed that the primary site in PR, alveolar RMS, M1 stage, tumor size more than 5 cm, IRS stage 4, surgery, radiotherapy, and chemotherapy were significant prognostic factors for OS (all *P* < 0.05), in which the primary site in PR, alveolar RMS, M1 stage, tumor size more than 5 cm as well as IRS stage 4 were associated with poor OS, while performing surgical, radiotherapy and chemotherapy treatment were associate with favorable outcome of OS. The results of multivariate Cox regression analysis showed that primary site in PR, alveolar RMS, M1 stage, IRS stage 4 were independent risk prognostic factors; surgery and chemotherapy were protective prognostic factors for HNRMS patients (**Figures 1A–H**).

**Table 3:** Univariate and multivariate Cox regression analysis of OS in head and neck rhabdomyosarcoma patients among children and adolescents.

Variables		OS Univariate		OS Multivariate	
		HR (95% CI)	*p*	HR (95% CI)	*p*
Gender					
	Male	Reference			
	Female	0.93 (0.59-1.48)	0.765		
Age		0.99 (0.94-1.05)	0.82		
Primary site					
	OR	Reference		Reference	
	PR	7.85 (3.24-19.03)	<0.001*	3.32 (1.09-10.13)	0.035*
	NOPR	3.52 (1.49-8.31)	0.004	1.46 (0.57-3.75)	0.427
Histology					
	Embryonal	Reference		Reference	
	Alveolar	2.67 (1.67-4.28)	0.001*	1.82 (1.10-3.02)	0.020*
	Other	0.64 (0.23-1.80)	0.398	0.82 (0.46-2.37)	0.715
T stage					
	T1	Reference			
	T2	0.84 (0.46-1.52)	0.565		
	Unknown	1.09 (0.60-1.96)	0.785		
N stage					
	N0	Reference			
	N1	0.85 (0.38-1.89)	0.693		
	Unknown	1.24 (0.74-2.09)	0.41		
M stage					
	M0	Reference		Reference	
	M1	8.23 (4.88-13.87)	<0.001*	2.20 (1.03-4.69)	0.041*
	Unknown	1.78 (0.95-3.34)	0.074	0.89 (0.37-2.14)	0.795
Tumor size					
	≤5cm	Reference		Reference	
	>5cm	3.99 (2.36-6.72)	<0.001*	1.53 (0.82-2.84)	0.180
	Unknown	1.39 (0.64-3.05)	0.404	1.22 (0.52-2.86)	0.649
IRS stage					
	1	Reference		Reference	
	4	8.35 (5.18-13.64)	<0.001*	2.53 (1.23-5.22)	0.012*
	Unknown	0.43 (0.13-1.41)	0.166	0.28 (0.07-1.04)	0.058
Surgery					
	No/Unknown	Reference		Reference	
	Yes	0.23 (0.13-0.41)	<0.001*	0.52 (0.27-0.99)	0.048*
Radiotherapy					
	No/Unknown	Reference		Reference	
	Yes	0.54 (0.33-0.88)	0.013*	1.42 (0.81-2.50)	0.221
Chemotherapy					
	No/Unknown	Reference		Reference	
	Yes	0.26 (0.13-0.49)	<0.001*	0.45 (0.22-0.93)	0.032*

* *P* value is significant.

### Construction and validation of nomogram model

**Figure 2:**
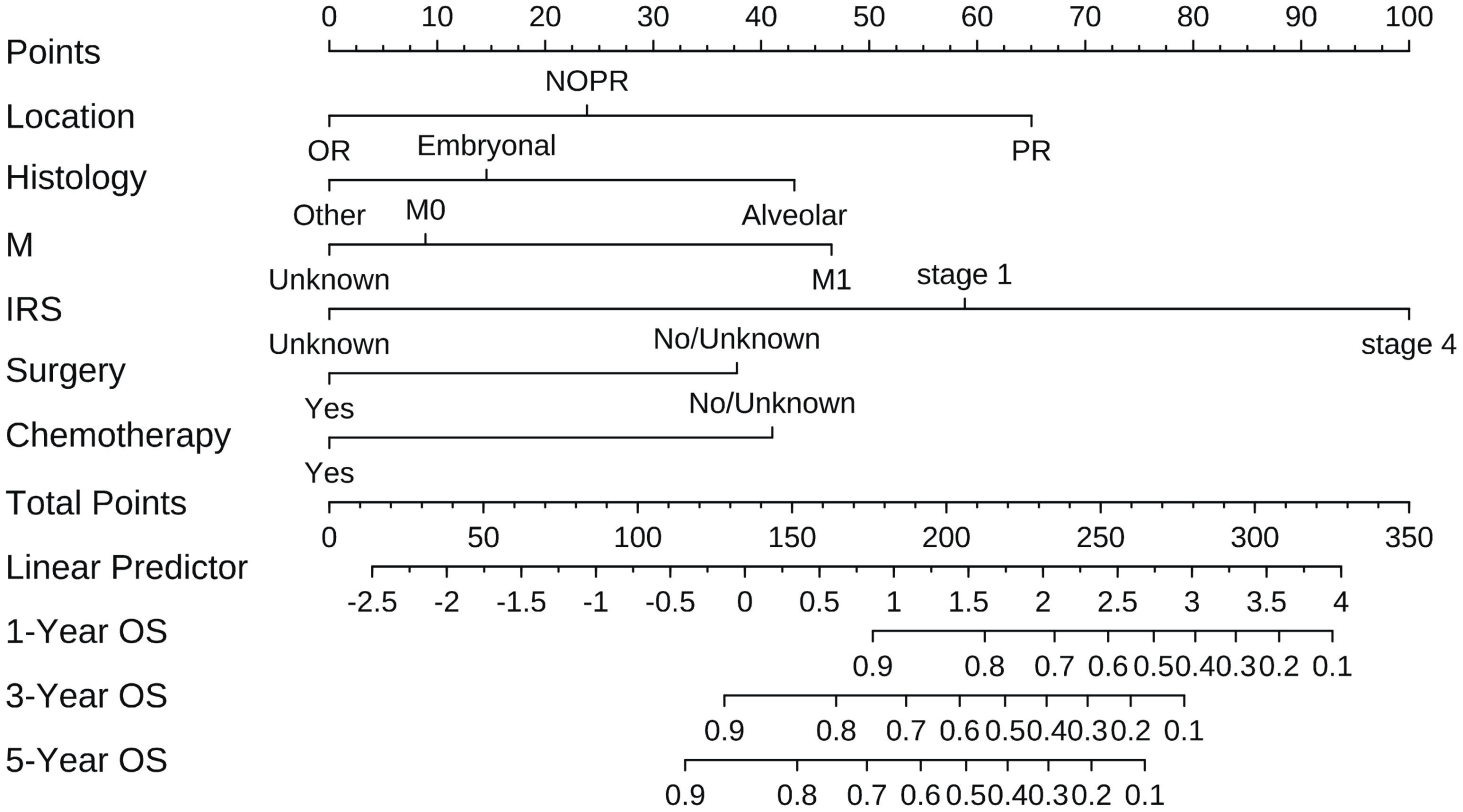
Prognosis model of 1-, 3-, 5-year OS in head and neck Rhabdomyosarcoma patients.

**Figure 3:**
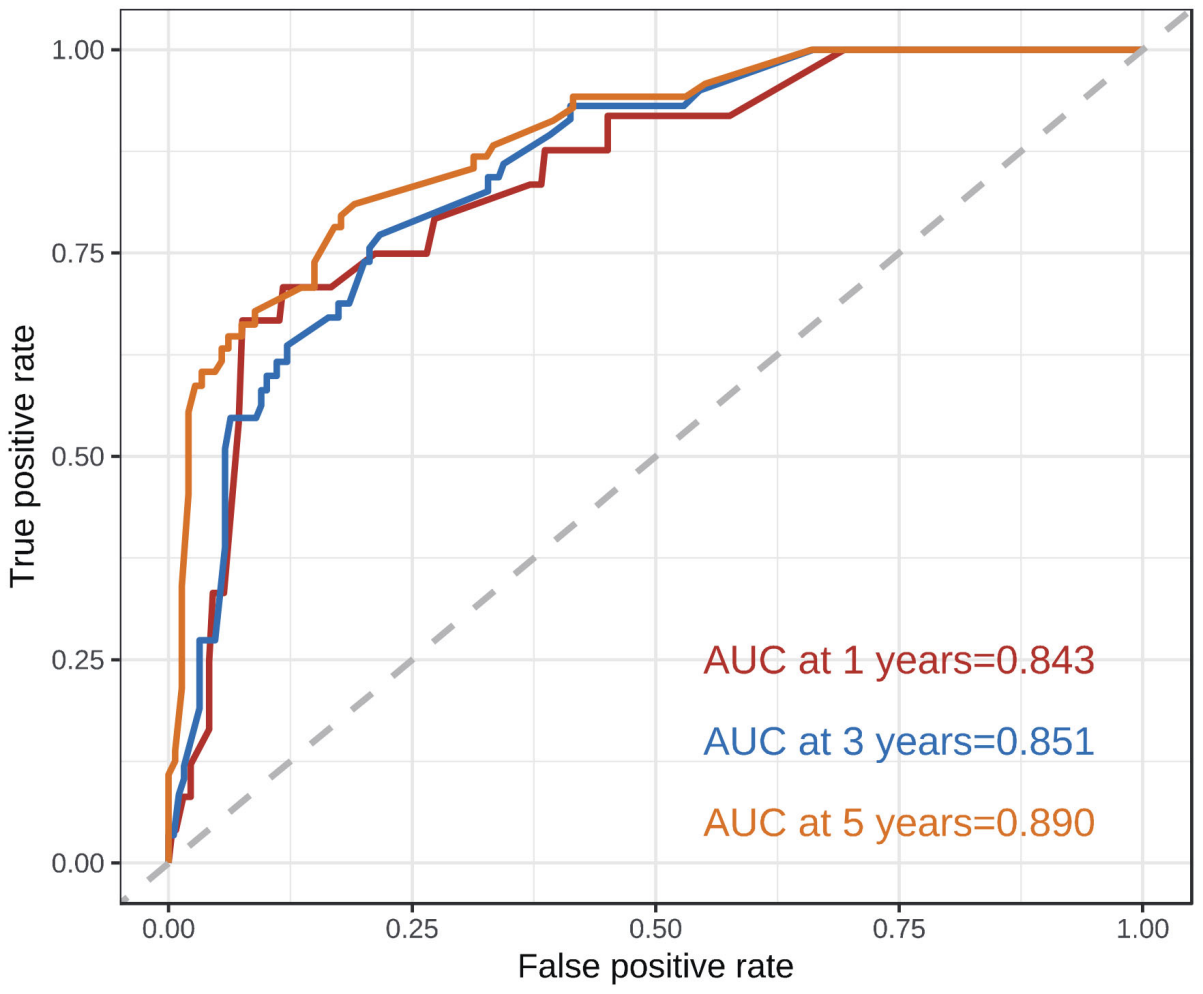
ROC curves of 1-, 3-, 5-year OS in HNRMS patients. HNRMS: head and neck rhabdomyosarcoma; OS: overall survival; AUC: area under the curve.

**Figure 4:**
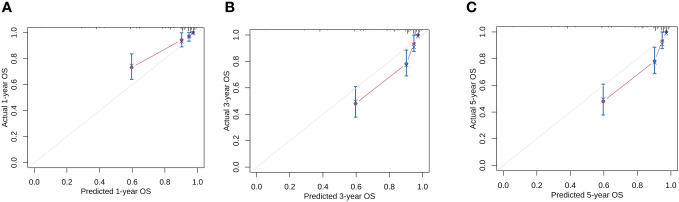
Calibration curves of the nomogram model for 1-, 3-, 5-year OS in HNRMS patients **(A–C)**. HNRMS, head and neck rhabdomyosarcoma; OS, overall survival.

**Figure 5:**
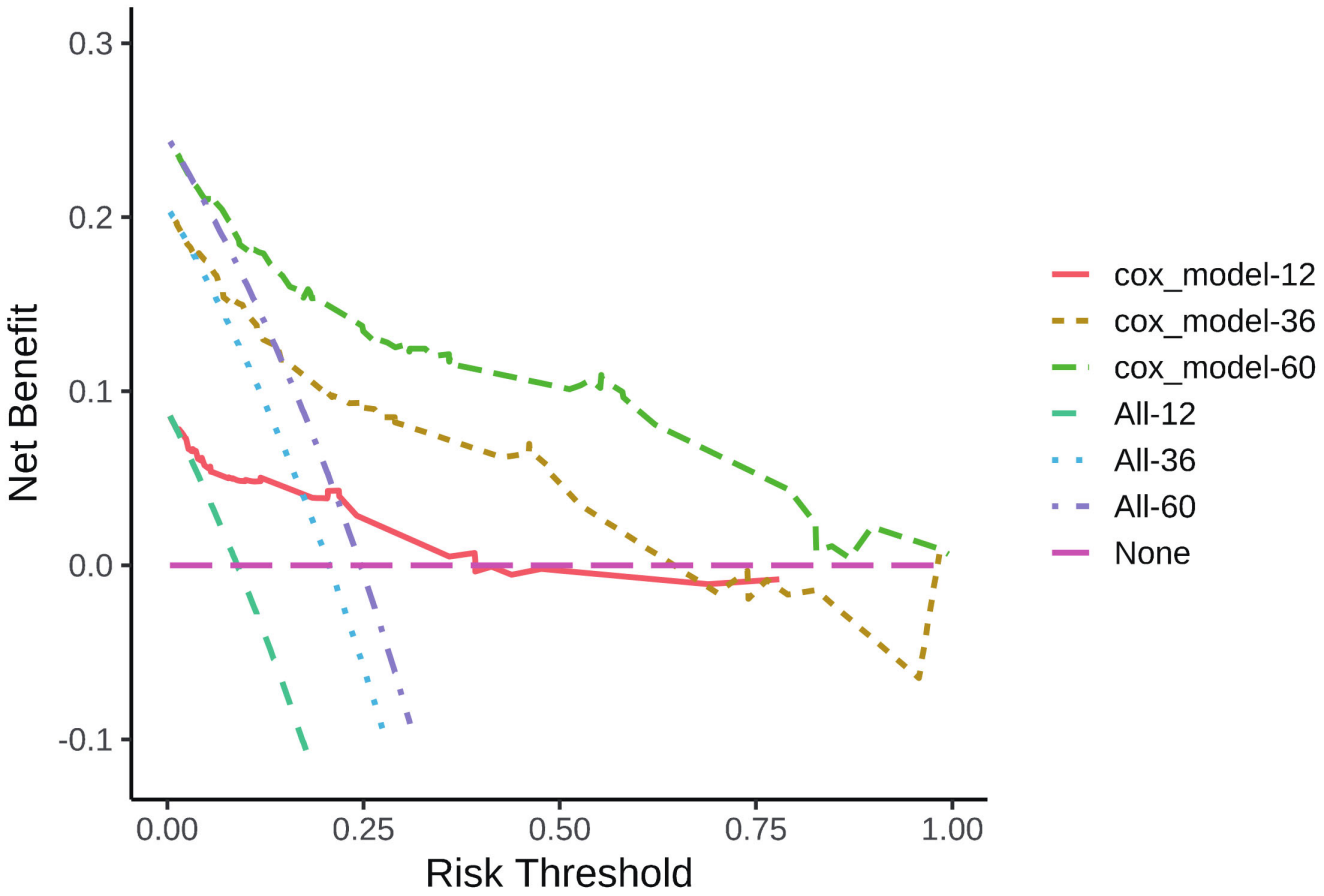
DCA curve of 1-, 3-, 5-year OS in HNRMS patients. HNRMS: head and neck rhabdomyosarcoma; OS: overall survival; DCA: decision curve analysis.

According to the results of multivariate analysis, tumor primary site, histology, M stage, IRS stage, surgery, and chemotherapy were associated with OS (all *P* < 0.05). We incorporated above six factors into constructing 1-, 3-, and 5-year nomogram of OS rate (**Figure 2**), and the point corresponding to each variable was projected to the scoring scale; finally, the scores of each variable were summed up to get the total scores. The AUC values of 1, 3, and 5 years OS were 0.843, 0.851, 0.890, respectively (**Figure 3**). The calibration curves shown in (**Figures 4A–C**) presented a good consistency between the nomogram predicted and actually observed 1, 3, and 5 years OS, which indicated that the predictive efficacy of the model was high. The decision curve analysis (DCA) was applied to render the clinical benefit value to the nomogram using the training cohort. The results showed that the prediction model had a high-clinical benefit value, good ability to assess the prognosis of patients with HNRMS, and good clinical practicability (**Figure 5**).

## Discussion

The tumor primary site was classified into favorable and unfavorable based on criteria used for staging pediatric tumors (21). In terms of HNRMS, favorable site refers to OR and NOPR, while unfavorable refers to PR. In our study, both univariate (HR: 7.85, 95% CI: 3.24–19.03, *P* < 0.001) and multivariate Cox regression (HR: 3.32, 95% CI: 1.09–10.13, *P* = 0.035) showed that PR was associated with poor OS, which is consistent with previous studies (22, 23). This may be due to the fact that the PR is close to the intracranial area and could not be completely removed (24). Furthermore, compared with superficial sites such as OR, PR is less likely to be detected, making it impossible to find and diagnose the tumor in time, and the lesion may have distant metastasis in early stage, which may metastasize first to the lungs or the bones (25). Orbital RMS affects eye movements at an early stage, so it is usually detected earlier and has a better prognosis.

In our study, the proportion of tumor size more than 5 cm was about 29.4%, which was lower than 51% in the IRS-IV study (9), 47.2% in the study of Ma et al. (26), and 75.5% reported by the Japanese researchers Hosoi et al. (27); this may be attributed to HNRMS limited growth space. Unsal et al. (28) and Dantonello et al. (29) reported that tumor size is a risk factor for poor survival. This is consistent with our study finding that a larger tumor was an adverse prognostic factor in HNRMS patients. Studied showed that tumor size with more than 5 cm was independent risk predictor for survival and local treatment failure (30); the result of univariate Cox regression showed that tumor size with more than 5cm was a risk factor for prognosis in children and adolescence (HR: 3.99, 95% CI: 2.36–6.72, *P* < 0.001).

RMS was divided into four subgroups: embryonal, alveolar, pleomorphic, and spindle cell/sclerosing RMS, according to the World Health Organization (WHO) classification system, of which embryonal was the most common subtype (31), embryonal RMS had a clearly more favorable clinical prognosis than alveolar type, which was observed to occur at an earlier age and associated with better prognosis (32, 33). Alveolar RMS was common in the muscle of the trunk and limb extremities and presented as a poor prognosis (32, 34, 35), studies showed that microarray-based gene expression profiling showed that PAX-FOXO1 fusion gene-positive alveolar RMS had a worse biological behavior than PAX-FOXO1 fusion gene-negative (36, 37). With this, it was evident that FOXO1 fusion status was an important prognostic factor and has replaced pathologic staging in RMS risk stratification in recent years (38, 39). In our study, univariate (HR: 2.67, 95% CI: 1.67–4.28, *P* < 0.001) and multivariate Cox regression (HR: 1.82, 95% CI: 1.01–3.02, *P* = 0.020) showed that alveolar RMS was associated with poorer OS than embryonal RMS, which is consistent with previous research (40).

Distant metastasis of RMS was found to be associated with worse survival; a study conducted by Turner and Richmon (41) found that patients with localized and regional disease had a higher 5-year survival rate than patients with distant metastasis (92.4%, 60.1%, vs. 36.6%), and the HR values were 0.15 and 0.37, respectively. Our results are consistent with Turner and his partner’ findings with the data of univariate Cox regression (HR: 8.23, 95% CI: 4.88–13.87, *P* < 0.001) and multivariate Cox regression (HR: 2.20, 95% CI: 1.03-4.69, *P* = 0.041). In addition, exiting literature suggested that tumors of parameningeal sites present advanced stage; they tended to invade distant tissues and organs (34, 42). As mentioned, embryonal RMS occurred with localized disease and in children at an earlier age. On the other hand, alveolar RMS presented most commonly with distant metastasis as well as in adolescents.

Surgical treatment was often consider as a critical treatment means for improving survival of RMS patients (43); surgeons tended to perform radical resection to remove tumor as well as its adjacent tissues and lymph nodes. However, expansion of the surgical scope in pursuit of negative margins would destroy important structures and functions for HNRMS in children and adolescents. Furthermore, tumor sites in dangerous site, such as intracranial extension, bony erosion of cranial base, or cranial nerve palsy, had increased tremendously surgical feasibility (9). The use of radiation and chemotherapy therapy was also found to be effective in improving survival (40, 41); studies showed that radiotherapy combined with chemotherapy is primary means for patients with unresectable primary tumors after initial treatment (44, 45). Our study showed that surgery, radiotherapy, and chemotherapy were all effective treatment methods for HNRMS in children and adolescences (*P* < 0.001, *P* = 0.013, *P* < 0.001, respectively).

There are a few limitations of this study. First, there are several editions of TNM stage, which could have an affection on IRS stage. Second, the SEER database lacks information on laboratory tests, specific radiotherapy and chemotherapy regimens, and comorbidities; however, they also play a critical role in prognosis of HNRMS (46, 47). Szkandera et al. (48) demonstrated a decreased lymphocyte/monocyte ratio (LMR) represents a novel independent poor prognostic factor in STS patients. Fausti et al. (49) also drew consistent conclusions that LMR is a specific predictor of Trabectedin efficacy and could be useful in daily clinical practice. Moreover, they highlighted a possible correlation between LMR levels and the percentage of intratumoral macrophages. Finally, this is a retrospective study, and the survival outcomes reported in SEER database are associated with different treatment guidelines, which are different from every era. Thus, selection bias could not be avoided.

## Conclusion

A prognostic nomogram model was constructed for predicting the probability of survival in HNRMS in children and adolescents, and this model presented practical and applicable clinical value to predict survival when choosing treatment strategies.

## Data availability statement

The original contributions presented in the study are included in the article/supplementary material. Further inquiries can be directed to the corresponding author.

## Author contributions

JW: Data curation, Software, Visualization, Writing – original draft, Writing – review & editing. QZ: Funding acquisition, Resources, Supervision, Writing – original draft, Writing – review & editing.
